# Perceptions of cell-cultured “meat” embedded in the cultural context: public surveys in Japan and the United Kingdom

**DOI:** 10.3389/fnut.2026.1756443

**Published:** 2026-02-10

**Authors:** Aiko Hibino

**Affiliations:** Institute of Future Science, Institute of Science Tokyo, Tokyo, Japan

**Keywords:** cell agriculture, cross-cultural consideration, cultivated meat, food technology acceptance, public perceptions, questionnaire survey

## Abstract

The labeling of cell-cultured products for food application as “meat” remains contentious, with public perceptions varying across cultural contexts. This study examined how individuals in Japan and the United Kingdom (UK) perceive cell-cultured products, particularly whether they should be called “meat.” An online survey was conducted with 1,200 respondents aged 20–59 years (600 in Japan and 600 in the UK). The questionnaire included items on agreement with calling cultured products as “meat,” willingness to try cultured meat, images associated with meat, views on life, dietary patterns, and interest in environmentally friendly foods. Multiple correspondence analyzes were performed to identify clusters of opinions. Respondents interested in environmentally friendly food were more likely to support labeling cultured products as “meat” and to express willingness to try them. In Japan, those who have the relational view on life were more likely to show strong willingness to try cultured meat, whereas in the UK, vegetarian respondents were more likely to reject it. The categorical boundaries for ambiguous cell-cultured products are co-occurring with a globally expanding framework of environmental consciousness; however, attitudes are also shaped by culturally specific factors, as demonstrated in Japan and the UK. These findings underscore the importance of cultural context in guiding communication.

## 1 Introduction

In recent years, cell culture technology producing food products using animal muscle cells—often termed cultured “meat”—has attracted increasing attention, while this technology is still at the research and development stage (1). Numerous social surveys across more than 30 countries have examined consumer acceptance of cultured “meat” (2) and sought to identify the factors that shape its acceptability. Recent reviews and empirical studies have clarified these determinants, with many highlighting *perceived unnaturalness* as a major influence (3–11). In addition to *perceived unnaturalness*, a meta-analysis identified several predictors of willingness to consume cultured “meat”: *perceived ethicality, disgust, taste, and safety* (12).

Public acceptance studies have generally assumed that products derived from cell culture technology using animal muscle cells are equivalent to conventional meat, with limited exceptions (1, 13–15). This assumption is problematic, first, because the material category of cell-cultured products using animal muscle cells is ambiguous in the biological sense, considering that meat produced through animal husbandry involves complex processes that differ from those used in cell culture (13, 14). Several studies have examined key aspects of cultured “meat,” including its technical characteristics and associated ethical issues (14). Other studies have addressed the ontological ambiguity of cultured “meat,” drawing on observational studies of scientific practices (16–18).

Second, the nomenclature of how the products derived from cell culture technology using animal muscle should be labeled is becoming a political issue (19–22). FAO uses “cell-based food” as a working terminology and experts in this area suggested the international harmonization of the terminology (23).

Third, labeling for emerging technologies should be carefully examined because it affects public perceptions and evaluations. Previous studies (24–27) have examined how different labels affect the evaluation of cultured “meat.” However, most of these have analyzed effects of the prefix, e.g., “*in vitro*” or “*cultured,*” and few have examined the opinions on the labeling using “meat.” A survey in France analyzing the relationship between public perceptions and dietary habits found that flexitarians did not accept the use of the term “meat” to describe cell-cultured product (28). In addition, the applications of cell culture technology are diverse, and restricting discussions to meat substitutes introduces bias by overlooking both the advantages and disadvantages of the broader range of potential uses (29).

The cultural context of meat is essential when examining the relationship between cell-cultured product and conventional meat. Previous studies (1, 9, 13–15) have shown that meat producing is embedded in culture. Another study introduced a whole–parts framework, emphasizing that the cultural significance of meat depends on the relationship between the whole animal and its parts (29). The social value of meat is embedded within cultural practices such as cutting methods and allocation rules within local communities. In contrast, the modern food system presents a major problem of separating the final product from the processes through which it is produced, by treating meat primarily as a commodity.

To understand the influence of the advent of cell-cultured products, it is necessary to examine not only whether the public is willing to consume them but also how they consider them. Whether cultured “meat” is called meat may depend on how people perceive meat in daily life. Based on these concerns, this study aims to clarify patterns of public perceptions of cultured “meat,” focusing on whether it may be called meat. We conducted a questionnaire survey in Japan and the United Kingdom (UK) to investigate how perceptions of cell-cultured products relate to cultural contexts. Specifically, we examined associations among variables such as whether cultured “meat” may be called meat, willingness to try cultured “meat,” images of meat and living things, eating habits, and environmental awareness, and identified patterns of public perceptions of cultured “meat” in relation to cultural perspectives. A hypothesis-generating approach was used because associations among these variables have not been fully examined, and assuming causal relationships would be inappropriate.

The reasons for considering Japan and the UK in this study are as follows. First, both countries have relatively advanced public funding in cell culture technology in science and industry, such as NAPIC and CARMA in the UK and NEDO biomanufacturing in Japan. Second, there is a contrast between the UK, which has a stronger carnivorous culture and higher expectations for green innovation, and Japan, which has a weaker carnivorous culture and lower awareness of green innovation. However, since this study uses hypothesis-generating analysis, its aim is not to make a rigorous comparison but to extract the countries’ distinctive characteristics.

## 2 Method

An online survey was conducted in December 2024 with 600 respondents in Japan and in January 2025 with 600 respondents in the UK. Respondents were aged 20–59 years old and were equally distributed by sex (300 males and 300 females) and by age group (150 each in their 20s, 30s, 40s, and 50s) (Supplementary Table 1). Participants were confirmed in advance in one of two gender groups (male, female), and one of four age groups (20s to 50s). They were recruited from monitors registered at Cross Marketing Group in Japan and its cooperative research company in the UK. Randomly selected monitors were invited to participate, and those who agreed completed the questionnaire. The quality of the respondents’ response was checked by trap questions in the questionnaire and periodic verification by the Cross Marketing survey company.

The same questionnaire items were used in both surveys. Some questions originally designed in Japanese were translated into English by a translation professional (Editage). The author checked whether the translated English text reflected the intent of the original Japanese text and made corrections. The corrected English text was further verified by a native English proofreader. A literal translation of cultured meat exists in Japanese (*baiyo-niku*), and this word was presented with a brief explanation of the producing process. Therefore, the possibility of misunderstanding the object as other food technologies, like genetically modified food, is quite low. There are no English terms that fully express the connotation of the Japanese word “inochi wo morau” in answering the image of meat, and English native speakers evaluated “receive the life” and “exchanging lives” to be a better expression for describing a part of the original nuance, respectively. This terminology will be examined through future qualitative research. Before the survey was conducted, the Research Ethics Committee of the Faculty of Humanities and Social Sciences, Hirosaki University, reviewed all the study materials and approved the study (No. 2024–12).

In this survey, we analyzed data on six items: willingness to try cultured meat, agreement with labeling the cell-cultured product “meat,” image of meat, views regarding life, dietary patterns, and interest in environmentally friendly foods (Table 1). This study used the question item asking about the willingness to try cultured meat (WTT) because it has been used in many questionnaires to measure individual positive attitudes toward it (12), and because the relationship with other variables can be clearly analyzed. However, as WTT asks the trial intention to taste it, it should be noted that WTT does not necessarily equate to the desire to eat the products regularly (30–32). We used the same categories as those applied in a previous study for agreement with labeling cultured “meat” meat, and dietary patterns (26).

**Table 1:** Simple distributions.

Questions	Categories	Label	JP (%, *N* = 600)	UK (%, *N* = 600)
Are you interested in environmentally friendly food?	I am strongly interested	IEF4	6.2	42.5
	I am somewhat interested	IEF3	41.3	42.5
	I am not very interested	IEF2	31.0	8.7
	I have no interest at all	IEF1	16.5	4.0
	I do not know	–	5.0	2.3
Let us know which eating habit is most applicable to you.	Veganism	–	0.8	3.5
	Vegetarianism	Veg	1.7	7.3
	Flexible consumption of meat	Flex	19.2	32.0
	No restrictions on eating meat	Nor	57.3	31.2
	Regular consumption of meat	Reg	21.0	26.0
Would you be willing to try cultured meat?	Definitely no	WTT1	23.5	14.3
	Probably no	WTT2	24.0	13.8
	Unsure	WTT3	30.2	18.0
	Probably yes	WTT4	17.2	31.8
	Definitely yes	WTT5	5.2	22.0
Do you think it is appropriate to call food produced by cell culture technology “meat”? Or do you think it is inappropriate	Appropriate to call such food “meat”	callMEAT	41.5	57.5
	Inappropriate call such food “meat”	notcallMEAT	58.5	42.5
Which of the two statements below is closer to your view on “life”? (A) Life is lived as an individual that exists independently from other individuals. (B) Life and non-life cannot be separated, and they are continuously connected^a^	I agree with A	Independent 2	19.3	29.8
	I somewhat agree with A	Independent 1	41.7	25.7
	I somewhat agree with B	Connected 1	31.0	28.5
	I agree with B	Connected 2	8.0	16.0
Which of the objects below is closer to your associated image when you heard of “meat” (meat to eat)? Please choose the one that is most applicable.	Animals (Cattle, chicken, etc.)	Animal	18.7	57.8
	Steak	Steak	47.5	24.5
	Meat juice of lean meat	Juice	10.3	7.3
	Delicious	Delicious	11.5	5.8
	Receive the life^b^	Receive	9.0	1.7
	I do not know	–	3.0	2.8

a This question item was set considering that it has been suggested that the view of life emphasizing a continuum between living and non-living things, which differs from the concept of life emphasizing individuality, is important for understanding the concept of “life” in the Japanese context (37).

b The survey might not have conveyed the exact nuances of the option “receive the life (as associated image with meat)” for the UK respondents due to insufficient translating from Japanese to English. It should be noted that this option may be measured differently in Japan and the UK.

Multiple correspondence analyzes were conducted using six categorical variables. Multiple correspondence analysis (MCA) is a multivariate method that analyzes multiple categorical measures simultaneously and identifies categories likely to be selected together, allowing the extraction of response patterns among respondents (33–36). This study adopted MCA for three reasons. First, because research on cultural aspects of attitudes toward cultured meat is limited, an exploratory analysis is more appropriate. Second, causal relationships among the study variables are not fixed. Although willingness to try cultured meat is often treated as an outcome variable, it may also influence the understanding of cultured meat. Therefore, an analysis based on correlations rather than predetermined causal directions is preferable. Third, MCA effectively handles categorical variables. Standard surveys often treat agreement levels as continuous variables, but the questionnaire scales used here were not equidistant, and differences in opinion characteristics across categories required examination. Additionally, prior research indicates no linear relationship between acceptance of cultured meat and perceived unnaturalness in Japan (37).

In this study, MCA was applied to response data from Japan and the UK. After excluding categories with a frequency below 5%, MCA was conducted on 23 categories in Japan and in the UK (Table 1). The highest explanatory value for similarity between categories was plotted as their distribution along the *x*-axis. In MCA, the proximity of plots A and B indicates that respondents who selected opinion A were more likely to select opinion B. Up to the fifth dimension was calculated, and we interpreted up to the third dimension with a cumulative contribution of more than 60%.

## 3 Results

**Figure 1:**
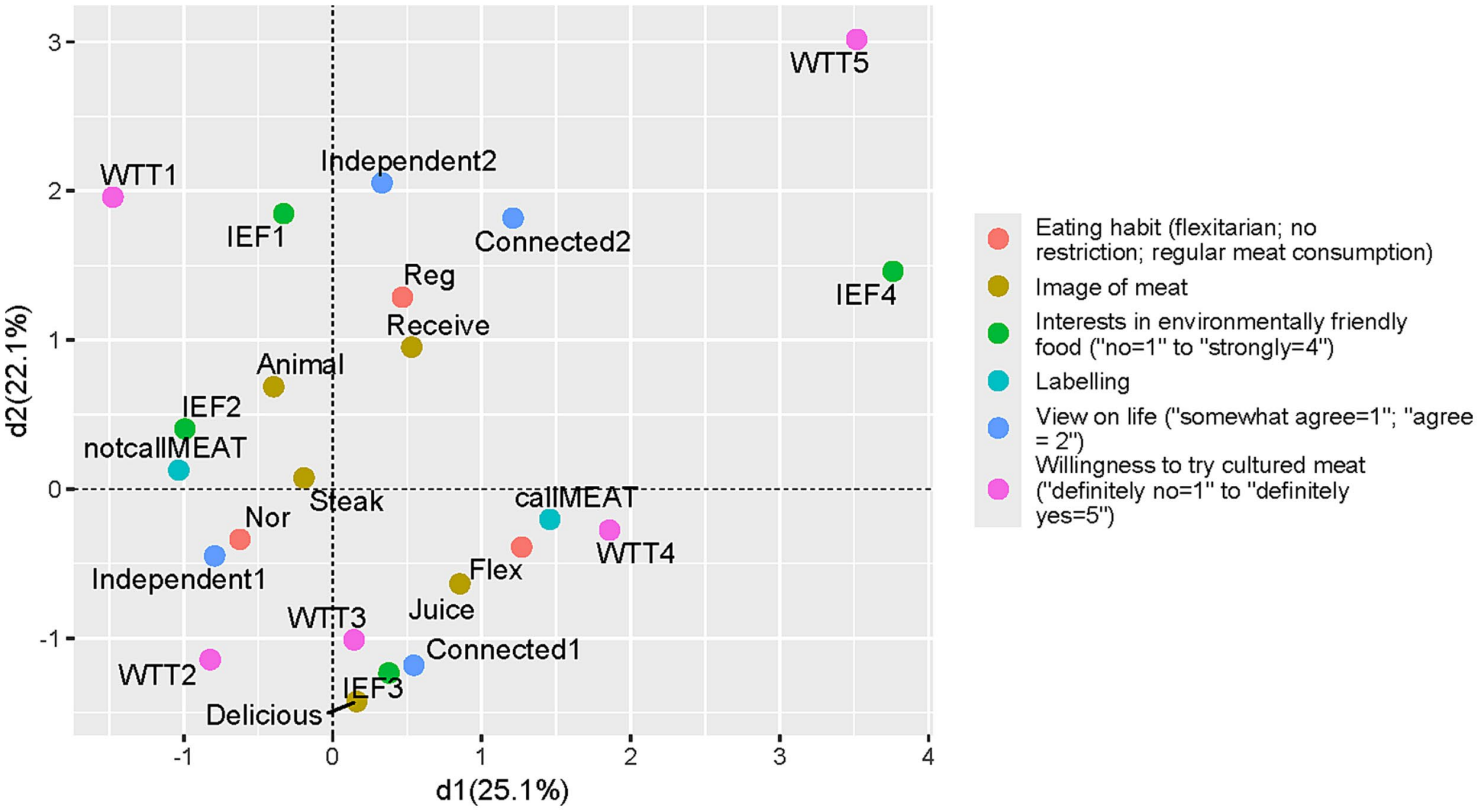
Pattern of attitudes toward the cell-cultured product in Japan (dimension 1 and 2).

**Figure 2:**
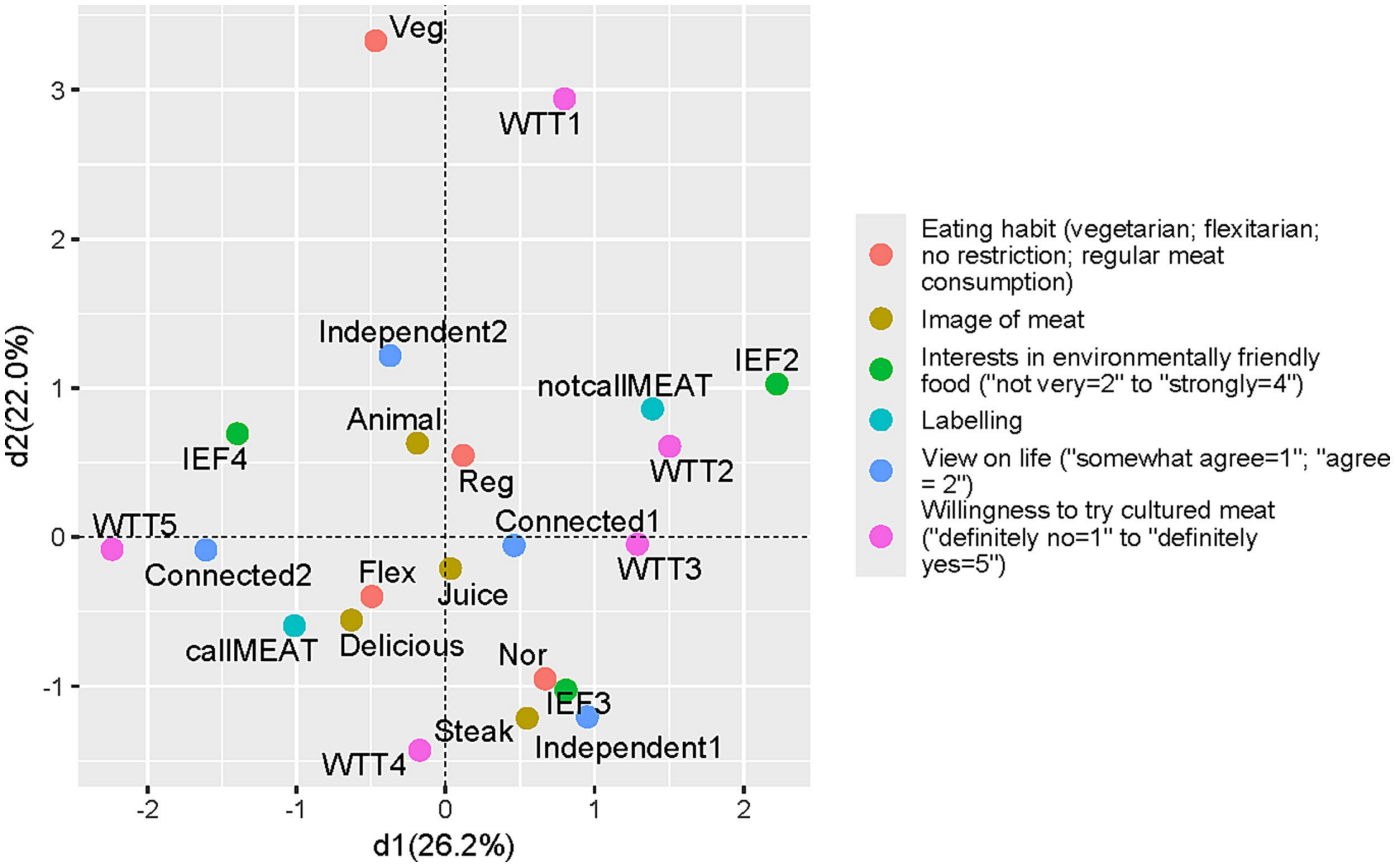
Pattern of attitudes toward the cell-cultured product in the UK (dimension 1 and 2).

In both the UK and Japan, the analysis shows clear differences in attitudes toward novel food between supporters and naysayers. Specifically, it distinguishes individuals with high environmental awareness, high receptivity in trying cultured “meat,” and agreement to label the cell-cultured product as meat from those with low environmental awareness, low receptivity in trying it, and disagreement with labeling the product as meat (Figures 1, 2). Figures 1, 2 present the highest explanatory power scores on the *x*-axis, and the second-highest scores on the y-axis.

In Figure 1 (Japan) and Figure 2 (UK), the categories “willing to try cultured meat: Definitely yes (WTC5) and Probably yes (WTC4)” were close to the category “interest in environmentally friendly foods: Strongly (IEF4)” and were also relatively close to the category “Appropriate to call such food “meat” (callMEAT).” Another cluster showed that “willing to try cultured meat: Probably no (WTC2)” was close to “interest in environmentally friendly foods: Not very (IEF2)” and “Inappropriate to call such food “meat” (notcallMEAT).” This differentiation in attitudes suggests that the narrative of global sustainability may play an important role in shaping perceptions of cell-cultured technologies and its products. It appears that cell-cultured food products have been approaching their social status as an alternative meat, successfully linked to strengthened environmental norms.

The pattern differentiation along the *x*-axis was similar in Japan and the UK; however, the pattern along the vertical (y) axis differed between the two countries. In Japan, a cluster of categories indicating strong opinions appears in the upper section, while intermediate opinions appear in the lower section (Figure 1). Previous Japanese social surveys have noted that clusters often reflect the strength of expression rather than the content of opinions. However, in the UK analysis, the categories “vegetarian (Veg)” and “Definitely no (WTC1)” were closely plotted, indicating that vegetarians in the UK tend to show lower interest in tasting cultured meat (Figure 2).

The differentiation in opinions on the MCA’s third dimension is notable, especially the contrast between Japan and the UK. Supplementary Figures 1, 2 present the third-highest explanatory power scores on the y-axis. In the Japanese analysis, responses indicating “the image of meat is to receive the life of animals (Receive)” appeared close to responses expressing relational views regarding life (Connected 2). These two categories were also positioned near the category of reflecting a strong interest in trying cultured meat (WTC5) in the third dimension (Supplementary Figures 1, 2).

## 4 Discussion

Our analysis indicates that individual perceptions of cell-cultured products align with the growing narrative of environmental concern in both Japan and the UK. The findings of this study can be developed into research on communication strategies. The meaning of cultured “meat” in an environmental context may appeal to those who already familiar with it, while it will be important to explain cell culture technologies outside of the meat category to those who hesitant about it.

Cultural context also plays a significant role. In Japan, we identified a small opinion cluster in which people who view cell-cultured products strongly positively tend to hold relational views on life and recognize that eating meat involves taking life. The recollection of connection between lives in relation to meat may be one of important factors in public understanding of emerging food technologies. In this concept, valuing the whole and part relationship of meat (29) as the essence of food leads to a favorable evaluation of decreasing excessive consumption. Cell cultured technology become to be regarded as the solution to this problem, while we need to carefully consider whether that technology is the only solution. In addition, it calls for further consideration on cultural specificity, taking into account the difficulties of translation.

In the UK, those who imagine animals as meat tend to hold more relational views on life. However, these views were closely associated with intermediate opinion in tasting cultured “meat,” indicating that views on life were not necessarily linked to positive or negative evaluation of cell-cultured products.

Environmental awareness and receptivity to cell-cultured products are more closely connected, with eating habits playing a significant role in the UK. Yet, the willingness to try cell-cultured products not directly proportional to meat consumption: daily meat eaters show moderate interest in tasting cultured “meat,” whereas vegetarians generally exhibit negative feelings toward it, which are considered to associate with the social identity (38).

This brief report examines the relationship between individuals’ general attitudes and lifestyles and their judgment of whether cultured “meat” qualifies as meat. One limitation of this study is its use of an exploratory analysis method. MCA extracts opinion patterns on a trial basis by simultaneously analyzing multiple factors of a subject with limited prior knowledge. Since we adopted an exploratory, hypothesis-generating approach, the results require further verification. Another limitation of this study is the representativeness of the sample. We should consider the bias of respondents in an online questionnaire survey. In addition, because respondents aged 60 years or older were not included in this study, the associated factors related to the perception of cultured “meat” in elderly people need to be examined separately. Nevertheless, we identified two distinct perspectives on whether cultured “meat” is considered meat and clarified public perception in identifiable patterns.

## Data Availability

The data supporting these conclusions will be made available by the authors upon reasonable request. Requests to access the datasets should be directed to AH.
