# Supplementary material for: Perceptions of cell-cultured “meat” embedded in the cultural context: public surveys in Japan and the United Kingdom
Source: Front Nutr. 2026 Feb 10;13:1756443. doi: 10.3389/fnut.2026.1756443 (PMC12929168; doi:10.3389/fnut.2026.1756443)
Supplement: Supplementary file 1 [file Table_1.docx]

**Supplementary files**

**Table I.** Socio-demographics of respondents

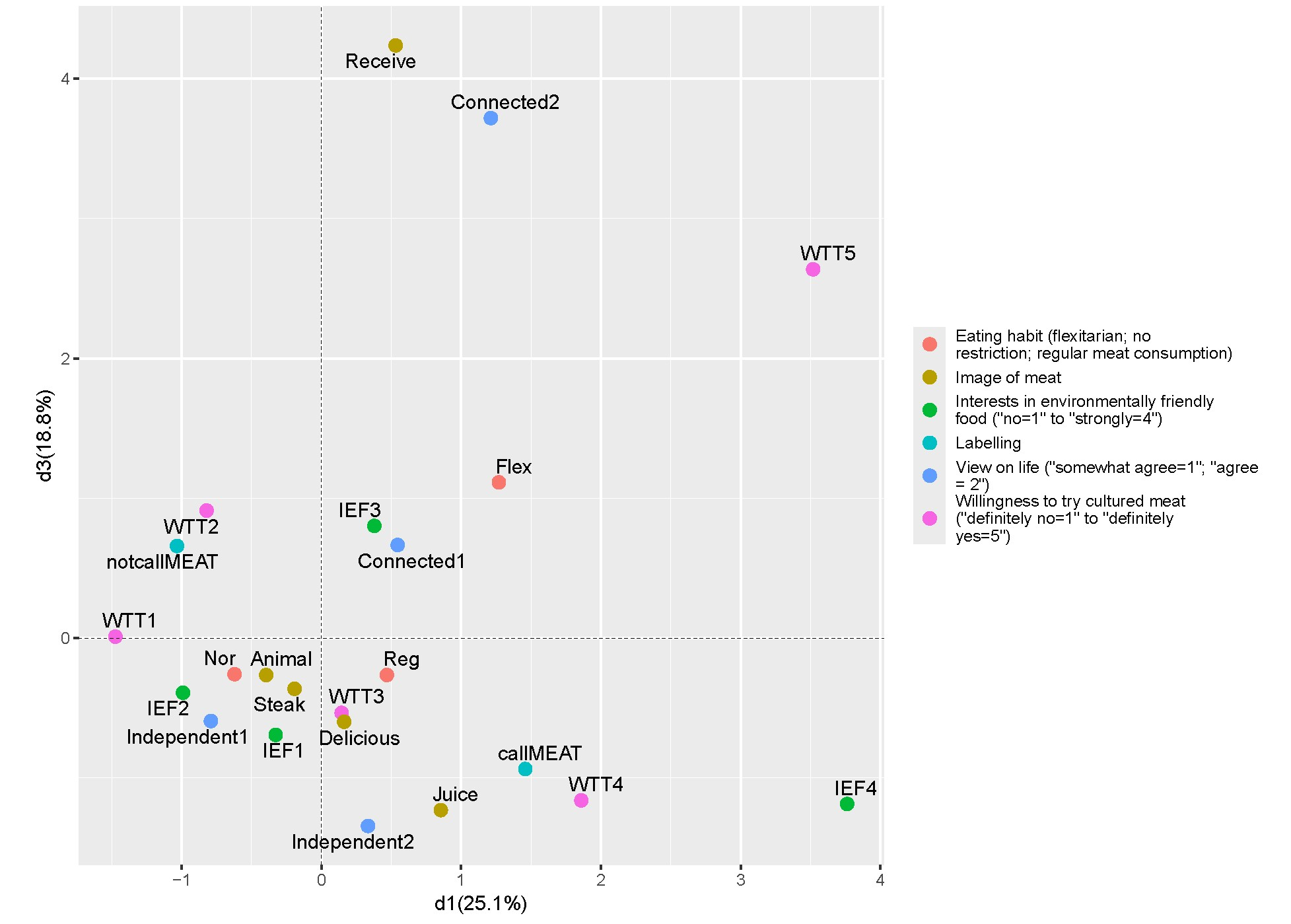


**Figure I.** Pattern of attitudes toward cell-cultured product for meat in Japan (dimension 1 and 3)


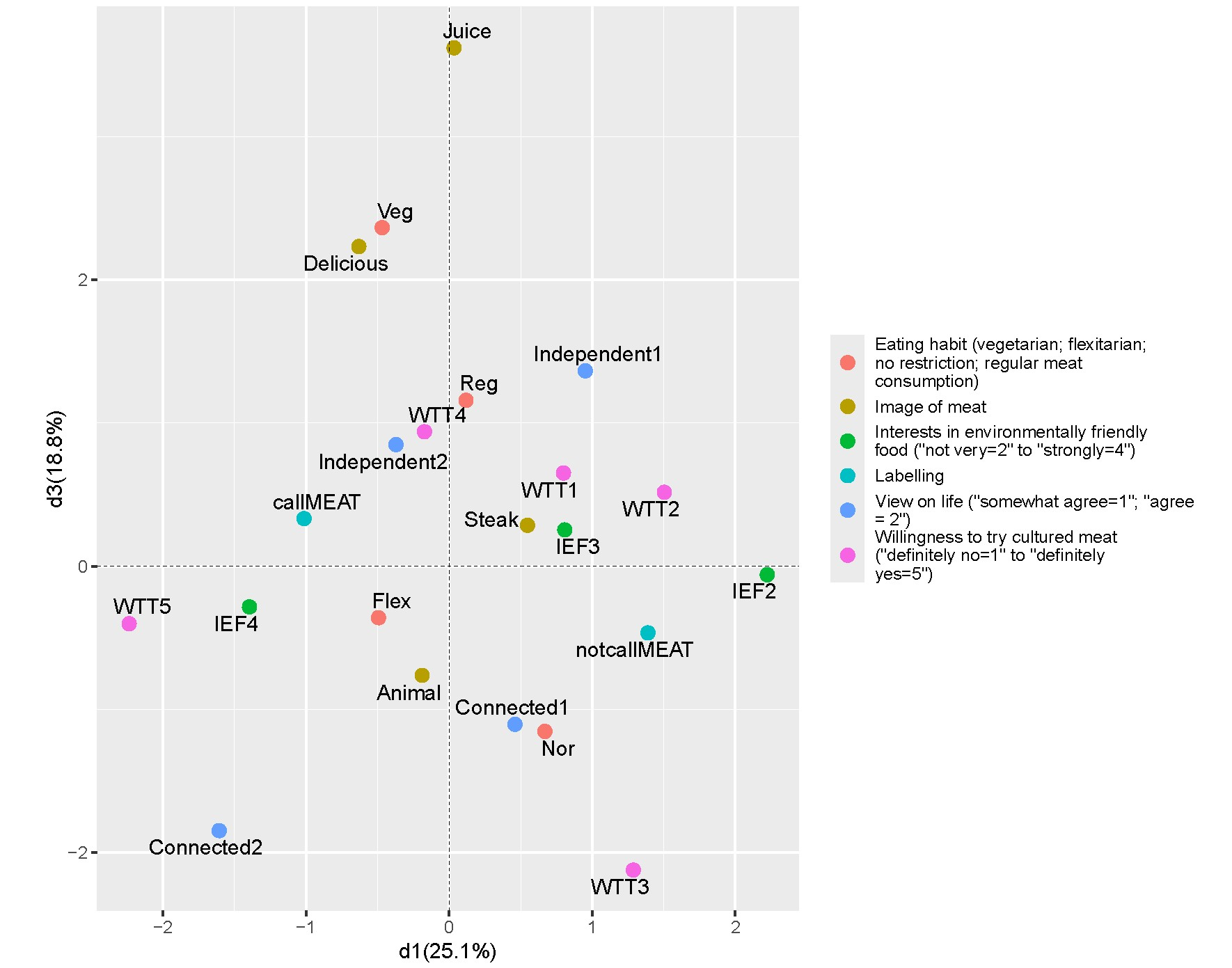


**Figure II.** Pattern of attitudes toward cell-cultured product for meat in the UK (dimension 1 and 3)
